# Comprehensive prediction of chromosome dimer resolution sites in bacterial genomes

**DOI:** 10.1186/1471-2164-12-19

**Published:** 2011-01-11

**Authors:** Nobuaki Kono, Kazuharu Arakawa, Masaru Tomita

**Affiliations:** 1 Systems Biology Program, Graduate School of Media and Governance, Keio University, Endo 5322, Fujisawa, Kanagawa 252-8520, Japan; 2 Institute for Advanced Biosciences, Keio University, Japan, Endo 5322, Fujisawa, Kanagawa 252-8520, Japan; 3 Department of Environment and Information Studies, Keio University, Endo 5322, Fujisawa, Kanagawa 252-8520, Japan

## Abstract

**Background:**

During the replication process of bacteria with circular chromosomes, an odd number of homologous recombination events results in concatenated dimer chromosomes that cannot be partitioned into daughter cells. However, many bacteria harbor a conserved dimer resolution machinery consisting of one or two tyrosine recombinases, XerC and XerD, and their 28-bp target site, *dif*.

**Results:**

To study the evolution of the *dif/*XerCD system and its relationship with replication termination, we report the comprehensive prediction of *dif *sequences *in silico *using a phylogenetic prediction approach based on iterated hidden Markov modeling. Using this method, *dif *sites were identified in 641 organisms among 16 phyla, with a 97.64% identification rate for single-chromosome strains. The *dif *sequence positions were shown to be strongly correlated with the GC skew shift-point that is induced by replicational mutation/selection pressures, but the difference in the positions of the predicted *dif *sites and the GC skew shift-points did not correlate with the degree of replicational mutation/selection pressures.

**Conclusions:**

The sequence of *dif *sites is widely conserved among many bacterial phyla, and they can be computationally identified using our method. The lack of correlation between *dif *position and the degree of GC skew suggests that replication termination does not occur strictly at *dif *sites.

## Background

In bacteria, replication fork arrest is mainly repaired by homologous recombination [1]. When such a recombination event occurs an odd number of times in one DNA replication event of circular chromosomes, the replicated chromosome is not properly segregated into two daughter chromosomes but instead produces a concatenated dimer [2,3]. Therefore, many bacteria harbor highly conserved chromosome dimer resolution (CDR) machinery to separate the dimer chromosome into two monomer daughter chromosomes.

In *Escherichia coli*, chromosome dimers are resolved by two tyrosine recombinases, XerC and XerD, by the addition of a crossover at a specific 28-bp sequence called the *dif *site, which is located in the replication termination region of the chromosome [4,5]. The *dif *sequence contains a pair of palindromic sequence motifs that correspond to the binding domains of XerC and XerD. The reaction is coordinated to the last stages of cell division by an essential cell division protein, FtsK, which functions as a septum-located DNA translocase [6-10]. FtsK moves along the chromosome unidirectionally towards the *dif *sequence, thanks to polar and orientated sequences, the KOPS [11-13]. CDR is initiated when FtsK reaches *dif *and its extreme C-terminal domain directly interacts with the C-terminal domain of XerD [14-18]. The *dif*/XerCD chromosome dimer resolution system seems widely conserved. *In vivo *experimental evidence for its conservation has been obtained in *Xanthomonas campestris*, *Caulobacter crescentus *and *Vibrio cholerae *[19-21]. *In vitro *characterization of Xer recombinases and *dif *sites has also been carried in *Haemophilus influenzae *and *Bacillus subtilis *[22,23]. However, the importance of *dif*/XerCD for the fitness of bacteria has only been demonstrated in *E. coli *and *V. cholerae *[20,24]. In some other bacteria, like *Lactococci *and *Streptococci*, chromosome dimer resolution is resolved by single tyrosine recombinases that act at specific *dif *site [25,26]. In this case, dimer resolution still depends on FtsK and *dif *is still located opposite the origin of replication between oriented polar sequences [27]. Several filamentous phages are known to hijack this site-specific recombination machinery of *dif*/XerCD for their integration into the host chromosome, containing pseudo-*dif *sequences within these phage genomes [28-34]. However, the *dif *sequence remains intact during such recombination process to ensure the integrity of chromosome dimer resolution machinery [35,36]. The *dif*-like sequences in phages often contain more variable central region that is longer than the canonical 6 bp [31,33,34], and the XerD binding arm is considerably degenerate [28].

Because there is only one origin of replication on bacterial circular chromosomes, replication generally terminates in a specific region of the chromosome. This can be followed by the existence of a GC skew on the two replichore arms of the chromosomes with a shift-point opposite the origin of replication [37]. Based on the observation that *dif *sites are generally located at or near the GC skew shift-point, Hendrickson and Lawrence proposed that replication might generally terminate at *dif*, which coordinate replication and chromosome dimer resolution [38]. In *E. coli*, the replication process usually terminates at a narrow region that includes approximately 5% of the genome length and is located directly opposite the replication origin [39-41]. This is partly due to the existence of the Tus/Ter replication fork trap [41]. *dif *is located within the replication fork trap but termination occurs precisely at the Tus site, not at *dif *[42] and *dif *is active when displaced outside of the replication termination region if it is still within the zone where KOPS converge [24]. Nevertheless, the lack of universal conservation of the Tus protein may suggest that replication terminated at *dif *sites until the relatively recent takeover by the Tus-Ter system [43]. We reasoned therefore that the comprehensive identification of *dif *sites and of their location with respect to the GC skew shift-point in hundreds of complete genomes might provide clues to the evolution of the CDR machinery and its possible link with the replication termination mechanism in bacterial species.

Prediction of the *dif *sequences has been reported by several groups with different approaches. Hendrickson and Lawrence showed that sequence skew can be used to predict the location of *dif *sites, and they identified putative *dif *sequences in 25 bacteria based on sequence similarity [38]. Le Bourgeois and colleagues reported a new type of tyrosine recombinase, named XerS, which is responsible for CDR in *Streptococci *and *Lactococci *and this recombinase targets a 31-bp sequence element named *dif*_SL _[25]. For comparison, they predicted *dif *sequences in 22 Firmicutes based on their similarity to that of *B. subtilis *with Megablast [44] and on the fact that the *dif *sequence occurs only once per genome. Val and colleagues identified that *V. cholerae *chromosome II, whose many features are plasmid-like, has an original *dif *sequence independently, and therefore it has FtsK-dependent CDR [20]. For this purpose, they predicted *dif *sequences in five α-Proteobacteria and ten β-Proteobacteria that harbour multiple chromosomes, and discussed a conserved FtsK-dependent CDR on multiple chromosomes based on the close relative distance of the position of *dif *sequences and the GC skew shift-points. Their prediction method is based on a HMMER [45] score (<1.0e-05) with a profile built from 27 aligned *dif *sequences in the largest chromosomes of γ-Proteobacteria species, with manual checking for 6-bp spacing between two XerC and XerD binding motifs.

Carnoy and Roten reported the most comprehensive predictions to date, identifying putative *dif *sequences in 204 chromosomes in 137 Proteobacteria strains, discussing the high conservation of *dif/*XerCD systems and the possible loss of *dif *sequences in endosymbionts, with suggestions for other CDR mechanisms [46]. Here, the prediction was based on BLAST searches and YASS alignment [47] with the *dif *sequences of *E. coli *and *B. subtilis*, and candidates were selected based on their proximity to the GC skew shift-points and a single occurrence per chromosome. Previous predictions were therefore limited to three bacterial phyla: Proteobacteria, Firmicutes and Actinobacteria.

To this end, we describe comprehensive predictions for *dif *sequences based on a machine learning approach, tracing the phylogenetic conservation patterns of XerCD recombinases and using an iterative hidden Markov modeling method. Furthermore, we observed the relationship between predicted *dif *sequence positions and GC skew shift-points, and investigated whether replication termination occurs at the *dif *site.

## Results

### Overview of *dif *sequence prediction

**Figure 1:**
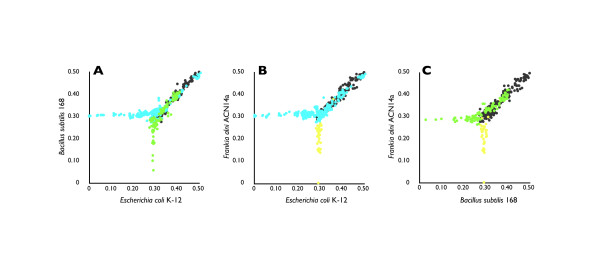
**The phylogenetic distance of XerCD in each organism**. The phylogenetic distances of bacterial genomes to three seed organisms, *Escherichia coli *(Proteobacteria), *Bacillus subtilis *(Firmicutes) and *Frankia alni *(Actinobacteria), were calculated as the average of phylogenetic distances of XerC and XerD. Detailed example is given in Additional file 1, Figure S1. A to C are scatter plots of the distances of these genomes to the seed organisms. Axes represent average distances as calculated by ClustalW. A, Distances from *Escherichia coli *K-12 and *Bacillus subtilis *168; B, distance from *Escherichia coli *K-12 and *Frankia alni *ACN14a; and C, distance from *Bacillus subtilis *168 and *Frankia alni *ACN14a. Blue represent the genomes of Proteobacteria, green represent Firmicutes, yellow represent Actinobacteria, and the gray marks represent other phyla. All phyla show strong preferences for seeds from the same phylum.

We first analyzed the phylogenetic conservation patterns of XerC and XerD in bacterial species by calculating the distances of their amino acid sequences from those in the seed organisms with known *dif *sequences (experimentally confirmed: *E. coli *and *B. subtilis *and computationally predicted: *Frankia alni*). As depicted in Figure 1 and Additional file 1, Figure S1, sequence similarity distributions were clearly distinguished by phylum. Sequences belonging to different phyla always showed ClustalW distances of ≥0.3, and based on this phylogenetic distribution pattern, we separately trained and predicted the *dif *sequences in each phylum using iterated HMM. By this phylogenetic prediction approach, we predicted *dif *sequences in 578 genomes out of 592 that harbor the XerCD recombinase (Additional file 2, Table S1 for a complete listing). The same prediction method was applied for 66 organisms with multiple chromosomes, totaling 142 chromosomes, where we could predict *dif *sequences in 63 organisms with 137 chromosomes (Table 1).

**Table 1:** Prediction result overview

Single Chromosome	Organism	Predicted	%
Proteobacteria	362	357	98.61
Firmicutes	100	97	97.00
Actinobacteria	66	66	100.00
Bacteroidetes	19	19	100.00
Chlamydiae	14	14	100.00
Chlorobi	11	11	100.00
Acidobacteria	3	3	100.00
Verrucomicrobia	3	3	100.00
Chloroflexi	3	3	100.00
Gemmatimonadetes	1	1	100.00
Nitrospirae	1	1	100.00
Elusimicrobia	1	1	100.00
Tenericutes	1	1	100.00
Spirochaetes	1	1	100.00
Cyanobacteria	5	0	0.00
Planctomycetes	1	0	0.00

Total	592	578	97.64

			

**Multiple Chromosomes**	Organism (chr)	Predicted (chr)	% (chr %)

Proteobacteria	60 (130)	57 (125)	95.00 (96.15)
Spirochaetes	6 (12)	6 (12)	100.00 (100.00)

Total	66 (142)	63 (137)	94.45 (96.48)

chr: chromosomes

All of these predictions resulted in unique hits above the threshold, and their validity was further confirmed through leave-one-out cross-validation. On the other hand, predictions below the threshold (score < 10 and e-value > 1.0E-04) often resulted in multiple candidates with insufficient scores. When the initial prediction using the strict threshold failed, we manually checked the predicted sequences for the conservation of palindromic structure in the 7-12-bp and 17-22-bp positions, and candidates that were located close to the origin of replication were removed because the displacement of a *dif *sequence near the origin significantly reduces the growth rate [24].

### Prediction results of each phylum

In Proteobacteria, fuzzy matching in 28 *Escherichia *strains based on the *dif *sequence of *E. coli *K12 for the creation of an initial seed profile hidden Markov model yielded a unique *dif *sequence in each of the 28 strains. Iterated HMM using this seed profile resulted in unique predictions over the validation threshold in 306 genomes. An additional 137 chromosomes in 69 genomes were predicted with iterated HMM separated by classes, and 10 distant genomes were predicted using an alternative seed profile created with the 3 most similar genomes. The predicted *dif *sequences totaled 482 in 414 organisms, with a prediction rate of 98.61% for single-chromosome strains and 95.00% for multiple-chromosome strains. Predictions failed in eight organisms and ten chromosomes, namely, *Agrobacterium tumefaciens *str. C58, *Paracoccus denitrificans *PD1222 chromosome I, II (α-Proteobacteria), *Burkholderia phytofirmans *PsJN chromosome I, *Burkholderia *sp. 383 chromosome I, III, *Nitrosospira multiformis *ATCC 25196 (β-Proteobacteria), *Desulfotalea psychrophila *LSv54 (δ-Proteobacteria), *Sulfurimonas denitrificans *DSM 1251 and *Nitratiruptor *sp. SB155-2 (ε-Proteobacteria).

For Firmicutes, fuzzy matching in 17 *Bacillus *strains (based on the *dif *sequence of *B. subtilis *str. 168 for the creation of the initial seed profile hidden Markov model) yielded a unique *dif *sequence in each of the 17 strains. Iterated HMM using this seed profile resulted in unique prediction over the validation threshold for 79 chromosomes in 79 genomes. The *dif *sequences are predicted in a total of 97 organisms, with a prediction rate of 97.00%. Prediction failed in three genomes, namely, *Clostridium perfringens *str. 13, *C. beijerinckii *NCIMB 8052 (Clostridia), and *Lactobacillus helveticus *DPC 4571 (Lactobacillales).

Although no experimentally confirmed *dif *sequence is available for Actinobacteria, that of *F. alni *is suggested to be 5'-CACGCCGATAATGCACATTATGTCAAGT-3' [38]. Therefore, we used this sequence for fuzzy matching in two genomes, *Nocardia farcinica *IFM 10152 and *Mycobacterium avium *subsp. paratuberculosis K-10, whose XerCD amino acid sequences were most similar to those of *F. alni*. Iterated HMM using this seed profile resulted in successful predictions above the validation threshold in all 66 genomes.

In Chlorobi, an initial seed profile was created with predicted *dif *sequences in *Chlorobaculum parvum *NCIB 8327 and *Prosthecochloris aestuarii *DSM 271 that scored above the validation thresholds using the Firmicutes profile, which resulted in the highest scores compared to those of Proteobacteria and Actinobacteria. Likewise, the profile of Firmicutes yielded the highest scores in Chlamydiae, where the initial seed profile was created from predicted *dif *sequences in *Chlamydophila pneumoniae *CWL029 and *Protochlamydia amoebophila *UWE25, which were below the validation thresholds, but contained palindromic structure and were located within 0.01-1.48 degrees from the shift-points of GC skew. Using these seed profiles, iterated HMM successfully predicted *dif *sequences in all 11 genomes in Chlorobi and 14 genomes in Chlamydiae.

Because the number of genomes is very small in all of the other phyla, we utilized the profiles of Proteobacteria, Firmicutes, Actinobacteria, Chlorobi, and Chlamydia that were created thus far instead of applying iterated HMM based on specific seed profiles, and all of the following candidates were confirmed based on scores, palindromic structure, and position. In Elusimicrobia and Tenericutes, all profiles showed high HMMER scores, and predictions using the profiles of Firmicutes and Chlamydiae predicted identical *dif *sequences. Similarly, the profiles of Firmicutes, Chlamydiae, and Proteobacteria predicted identical *dif *sequences in Nitrospirae, and predictions based on the profiles of Proteobacteria and Chlorobi were identical in Gemmatimonadetes.

In Spirochaetes, predictions using the profiles of Firmicutes, Chlamydiae and Proteobacteria profiles resulted in unique *dif *sequences in species with single chromosomes, and the profiles of Firmicutes were used for the predictions of 12 chromosomes in 6 species with multiple chromosomes, all with HMMER scores above the validation thresholds. The most suitable profiles varied among species in other phyla. In Acidobacteria, the *dif *sequence of *Acidobacterium capsulatum *ATCC 51196 was predicted by the profiles of Firmicutes, Chlamydiae, and Chlorobi *dif *sequences, and other species were predicted using the profile of Firmicutes only. In Verrucomicrobia, profiles based on Proteobacteria, Firmicutes and Chlorobi predicted *Methylacidiphilum infernorum *V4, and that of Proteobacteria and Firmicutes predicted *Opitutus terrae *PB90-1 and *Akkermansia muciniphila *ATCC BAA-835. In Chloroflexi, the Chlorobi profile was suitable for *Dehalococcoides *sp. BAV1 and *Dehalococcoides *sp. CBDB1, and that of Actinobacteria was used in *D. ethenogenes *195 *dif *sequences. *dif *sequences were predicted in 14 Bacteroidetes strains using the profile of Proteobacteria, and those in five strains were predicted using alternative profiles created with the three most similar genomes. In this way, we successfully predicted *dif *sequences in most phyla, although the prediction failed in the phyla Cyanobacteria and Planctomycetes.

### Correlation of the *dif *sequence position and the GC skew shift-points

**Figure 2:**
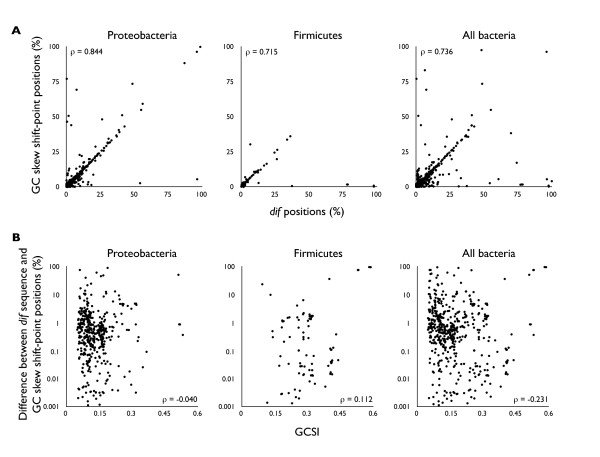
**The relationship between *dif *sites and GC skew**. A. Correlation of the GC skew shift-point (corresponding to the replication terminus region, Y-axis) and the locations of *dif *sequences (X-axis) for genomes with predicted *dif *sequences. Genomes with no visible GC skew, as indicated by GC skew Index (GCSI) ≤ 0.05, are omitted. Both axes are shown as the relative distance in percentage of half of the genome size (replichore size), from the position directly opposite of the replication origin. For example, 0% means that the position is directly opposite of the replication origin identified by the GC skew shift-point, and 100% means that it is at the replication origin. In other words, the higher the percentage, the closer the distance to the replication origin. Here the positions of GC skew shift-points and *dif *sites are strongly correlated in all three phyla. B. Lack of correlation between the difference in the positions of GC skew shift-points and *dif *sites (Y-axis) and the GCSI (X-axis). GCSI is a quantitative measure of the degree of GC skew, where GCSI = 0 is no observable skew, and GCSI = 1 is extremely pronounced skew. Typically GC skew is visible at GCSI ≥ 0.1, and it is pronounced when GCSI ≥ 0.3. Since we see no correlation in these plots, stronger replication-related mutation bias (i.e. larger GCSI) does not necessarily result in closer positions of the GC skew shift-point and the *dif *site. These results suggest that the replication termination occurs near the *dif *site, but not at the *dif *site. The number of *dif *sites is 517 in all bacteria, 438 in Proteobacteria and 97 in Firmicutes. The ρ in this figure is Spearman's rank-correlation coefficient.

Using the predicted *dif *sequences, we compared their positions within the genome to the shift-points of the GC skew. Firstly, we analyzed the distributions of relative genomic distances of *xerC*, *xerD *and *ftsK *genes from the predicted *dif *sites. As a result, *xerC *genes were mostly located near the *dif *sites, *xerD *genes were near the replication origin, and *ftsK *genes were located mostly in between *xerC *and *xerD *genes (Additional file 1, Figure S2). The comparison of positions between predicted *dif *sites and the shift-points of the GC skew showed that the *dif *sequences predicted in the phyla Proteobacteria and Firmicutes correlated significantly with the GC skew shift-points that are highly likely to be located within the terminus region (Spearman's rank correlation coefficients: ρ = 0.844 and 0.715, respectively; Figure 2A). The differences among these positions fell to within 0.00-1.39% of the genome for ±1σ, and outliers did not exceed 3% in distance relative to the genome size (Additional file 1, Figure S3). The above results confirm that chromosome replication and CDR are related, and that show the accuracy of the predictions described in this work.

To further investigate whether replication terminates at the *dif *site, by observing the overall contribution of the genomic selection/mutation pressures of the replication machinery to the collinearity of the *dif *sequence positions and GC skew shift-points, we plotted the distances between them against the GC Skew Index (GCSI) of genomes to quantify the degree of replicational mutation/selection pressures. GCSI is an index that quantifies the degree of GC skew of a given genome, which can be used as a comparative measure of the accumulated replicational mutation/selection pressures [48]. Since the strength of the GC skew is speculated to partly correlate with the growth rate of bacteria [49], high replication mutation/selection rate indicated by GCSI implies a greater number of replication events in these organisms. Therefore, if the replication terminates at or around the *dif *site, even allowing for statistical fluctuations, we can assume that the increasing number of replication events should shape GC skew shift-points closer to the *dif *site by the central limit theorem and by the law of large numbers. Hence, genomes with higher GCSI should have closer relative distance between the GC skew shift-points and *dif *sites, if replication terminates at the *dif *site. However, as depicted in Figure 2B, we observed no correlation between these two variables (Spearman rank correlation coefficients in Proteobacteria and Firmicutes: ρ = -0.046 and 0.112, respectively).

## Discussion

In this study, we first demonstrated that the conservation of XerCD genes follows phylogenetic conservation patterns that are specific to each bacterial phylum (Figure 1). Based on this principle, we comprehensively predicted the *dif *sequences in hundreds of completely sequenced genomes using a recursive strategy that iteratively models and predicts these sequences using profile hidden Markov models. As a result, we obtained unique candidate *dif *sequences in 715 chromosomes in 641 strains that were validated through multiple means, resulting in the largest collection of predicted *dif *sequences assembled to date. In comparison to previous work by Carnoy and Roten, which predicted *dif *sequences in 228 genomes, our predictions coincided with their results in 208 genomes and we added 507 genomes, including *Aromatoleum aromaticum *str. EbN1, which Carnoy and Roten reported to lack the *dif*/XerCD system. Excluding strains or chromosomes we could not predict, namely, *A. tumefaciens *str. C58, *Burkholderia *sp. 383 chromosome I, II, *D. psychrophila *LSv54, *N. multiformis *ATCC 25196, *P. denitrificans *PD1222 chromosome I, II and *S. denitrificans *DSM 1251, the predicted *dif *sequences in this study differed in 12 chromosomes in comparison to the results of Carnoy and Roten: *C. crescentus *CB15, *Granulibacter bethesdensis *CGDNIH1, *Pseudoalteromonas haloplanktis *TAC125 chromosome II, *Ralstonia eutropha *H16 chromosome II, *Rhodobacter sphaeroides *2.4.1 chromosome I, *R. sphaeroides *2.4.1 chromosome II, *Rickettsia bellii *OSU 85-389, *R. conorii*, *R. felis *URRWXCal2, *R. prowazekii*, *R. typhi *Wilmington, and *Shewanella *sp. ANA-3. For *R. eutropha *H16 chromosome II and *P. haloplanktis *TAC125 chromosome II, both studies predicted positions that were symmetric from the origin of replication, and although experimental confirmation is required to confirm which candidates function *in vivo*, the palindromic structures of the XerCD binding sites are more conserved in the candidates predicted by our method. Therefore, overall, our results were identical with those of Carnoy and Roten for 92% of the genome analyzed (208/228), and 11/12 mismatch resulted in candidates with more conserved XerCD binding sites, with the addition of 507 genomes among numerous phyla. Carnoy and Roten noted that some *Vibrio *species contain two *dif *sites both located at the vicinity of the GC skew shift-points. Therefore, we further tested whether the predicted *dif *sites in multiple chromosomes are all located near the GC skew shift-points. Using 5% genomic distance as a threshold, 45 out of 54 strains with two chromosomes, including *Vibrio *species, and 6 out of 9 strains with three chromosomes showed such agreement of the positions, (Additional file 2, Table S1).

There are four factors that may explain the advantages of our results. First, the selection of bacterial strains in the study by Carnoy and Roten was limited to genomes harboring XerCD that were identified by their similarity to those of *E. coli*, whereas we used all genomes with XerCD orthologs as identified by the KEGG Orthology database. While there is a little time-delay until the sequences are annotated and incorporated into the KEGG Orthology database, use of this database provides a more generic and comprehensive starting point. Second, similarity searches using software tools such as BLAST are not suitable for short sequence motifs that undergo mutation, and the difficulty in identifying only those *dif *sequences with sequence similarity has been shown for *C. crescentus *[50] and several classes of Proteobacteria [20]. Third, *dif *sequences require two binding motifs of XerC and XerD to be functional [51]; therefore, the conservation of palindromic structure at the 7-12-bp and 17-22-bp positions should be confirmed for each predicted candidate. Finally, the use of iterated HMM allowed *dif *sequence prediction using the profiles of closely related species for each iteration, following the phylogenetic conservation pattern of XerCD.

The high predictability shown in this study suggests that the *dif/*XerCD system of chromosome dimer resolution is highly conserved among bacterial species and that *dif *sequences are almost always conserved when XerCD is present within the genome. In fact, according to the KEGG Orthology database, XerC and XerD are conserved in approximately 60-70% of bacterial species, which is a higher percentage than is found for the replication termination protein Tus [52] and for universal genes such as the SOS response repressor LexA [53]. In light of the remarkable conservation of the *dif/*XerCD system, although it is beyond the scope of this study, explorations of alternative CDR machinery in species that lack the *dif/*XerCD machinery would be an interesting area of future research. Chromosome dimer resolution pathways are suggested to be present in species that lack the *dif/*XerCD system, and several alternative pathways have been reported and suggested. Le Bourgeois *et al*. reported an unconventional CDR pathway involving only one recombinase (XerS) in *Streptococci *and *Lactococci*, along with a 31-bp *dif *sequence [25]. Similarly, through computational analysis, Carnoy and Roten suggested the existence of another pathway, termed XerH, in ε-Proteobacteria in place of XerCD and XerS and discussed the likelihood of the existence of *dif *analogues in these species [26,46]. The basic strategy of iterated HMM should be applicable in predicting *dif *analogues in these species when defined seed sequences and detailed positions of recombinase binding sites are elucidated.

**Figure 3:**
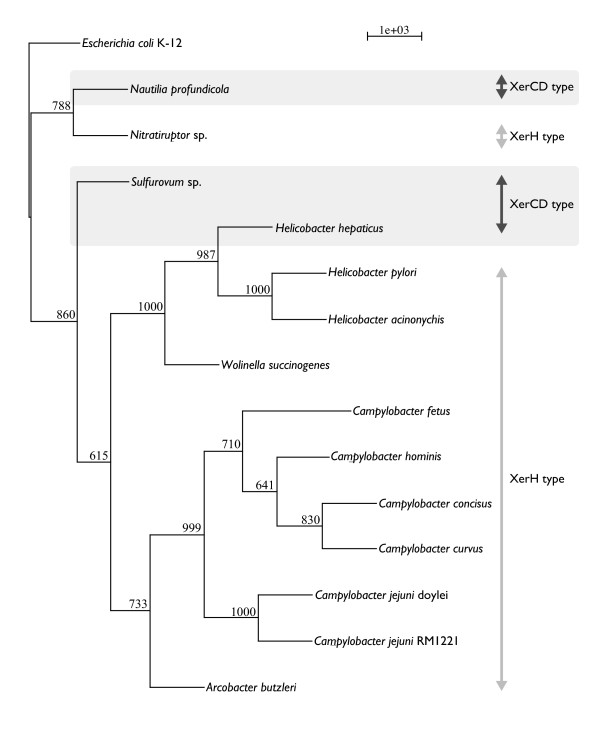
**Phylogenetic tree based on rRNA for the comparison of XerCD- and XerH-containing genomes**. This phylogenetic tree is constructed using the maximum-likelihood method and is based on 16S rRNAs of 14 organisms in ε-Proteobacteria, whose *dif *sequences are predicted in this study. The outgroup is *Escherichia coli *K12.

Although we limited our analysis to strains containing XerCD orthologs, our predictions failed in several species. In Proteobacteria, we could not identify *dif *sequences in five organisms and seven chromosomes, including species with single chromosomes (*Nitratiruptor *sp. SB155-2 and *S. denitrificans *DSM 1251) that are ε-Proteobacteria, where an alternative CDR mechanism involving XerH is suggested [46], and species with multiple chromosomes (*P. denitrificans *PD1222 chromosome I, *P. denitrificans *PD1222 chromosome II, *B. phytofirmans *PsJN chromosome I, and *Burkholderia *sp. 383 chromosome I and III). Among these, *B. phytofirmans *PsJN and *Burkholderia *sp. 383 contained *dif *sequences in other chromosomes, indicating that the *dif/*XerCD system is conserved in these strains. Similarly, in Firmicutes, we could not determine *dif *sequence in *L. helveticus *DPC 4571, *C. perfringens *str. 13 or *C. beijerinckii *NCIMB 8052. Among these strains, *L. helveticus *DPC 4571 has an alternative CDR recombinase XerS in its genome, indicating that the *dif/*XerCD system may not be functional. This is an intriguing example of possible evolutionary intermediate with the co-existence of two systems, presumably resulting from a horizontal gene transfer event. While we are unable to find a *dif *sequence corresponding to the XerS machinery, *xerS *gene in this species is located close to the GC skew shift-point (*xerC*: 1031814-bp, *xerD*: 1055574-bp, *xerS*: 1228715-bp, and GC skew shift-point: 1225733-bp), which is indicative of its functionality as shown in previous works [25,26,46]. *C. perfringens *str. 13 and *C. beijerinckii *exhibit highly biased GC contents (28.57% and 29.86%, respectively), and hidden Markov profiling of AT-rich *dif *sequences may have failed due to the background AT-richness of the genome. Comparative studies of *dif/*XerCD systems using close relatives of these genomes may provide evolutionary clues regarding the acquisition and loss of CDR machinery. For example, mapping the types of CDR machinery to the phylogenetic tree of ε-Proteobacteria obtained using 16S rRNA sequences with the dnaml program in the Phylip package shows that a XerH type of CDR machinery may have diverged at an early stage within this phylum. The XerCD type of CDR seems to be absent in the *Campylobacter *and *Helicobacter *genera, except for *Helicobacter hepaticus*, which suggests the existence of the XerH type of CDR in the common ancestor of these species (Figure 3). The *dif *candidate in *H. hepaticus *was predicted with iterated HMM only marginally above the threshold, with a score of 10.2 and an e-value of 5.5e-05. Further analysis is required to identify whether this species actually contains *dif/*XerCD or XerH-type machinery.

**Figure 4:**
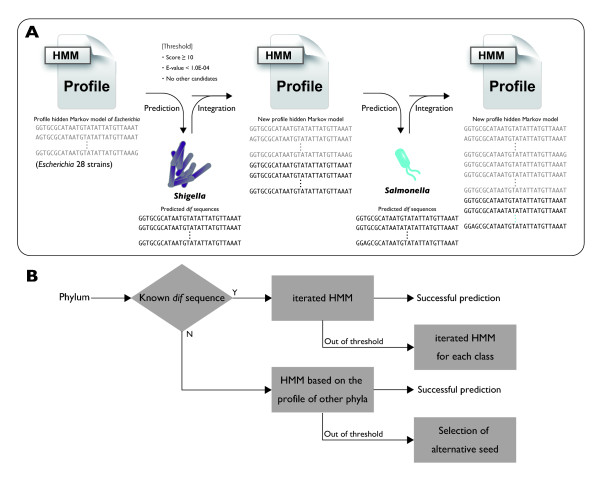
**Prediction strategy**. A. Example of the iterated HMM in Proteobacteria. The first seed profile hidden Markov model is created from the seed *dif *sequence of *Escherichia coli*, by searching for *dif *sequences in 28 genomes belonging to the genus *Escherichia *by means of fuzzy matching. Based on this initial profile hidden Markov model, *dif *sequences were predicted in the genomes of the closest genus to the *Escherichia *genus (in this case, *Shigella*) according to XerCD amino acid sequences. Subsequently, a new profile is created using the previous profile and the newly predicted *dif *sequences, and this new profile is used to predict in the second closest genus (in this case, *Salmonella*). In this way, profile creation and *dif *sequence prediction were repeated recursively in decreasing order of similarity of XerCD from the *Escherichia *sequence. In this way, iterated HMM is conducted for each phylum. B. Flow chart of the overall strategy.

Predictions failed in all species belonging to the phylum Cyanobacteria. Although XerCD is present in these species, the sequence similarity distance of XerCD in Cyanobacteria to those of other phyla was high (average of 0.358 ± 0.0159, N = 540), with a minimum distance of 0.322 to *Actinosynnema mirum *(Actinobacteria), which exceeded the 0.3 threshold that was shown in Figure 4. Therefore, this divergence of XerCD in Cyanobacteria from those of other phyla implies low applicability of the iterated HMM approach, which utilizes the phylogenetic conservation pattern of XerCD. One possible explanation for the prediction failure in this phylum is that the *dif *sequences and XerCD are highly divergent in Cyanobacteria, preventing their identification with sequence profiles. The replication origin in Cyanobacteria is yet to be identified, and GC skew is weak in these species, implying low degree of replicational mutation/selection pressures, which could also be a reason for the failure of prediction in these species.

**Figure 5:**
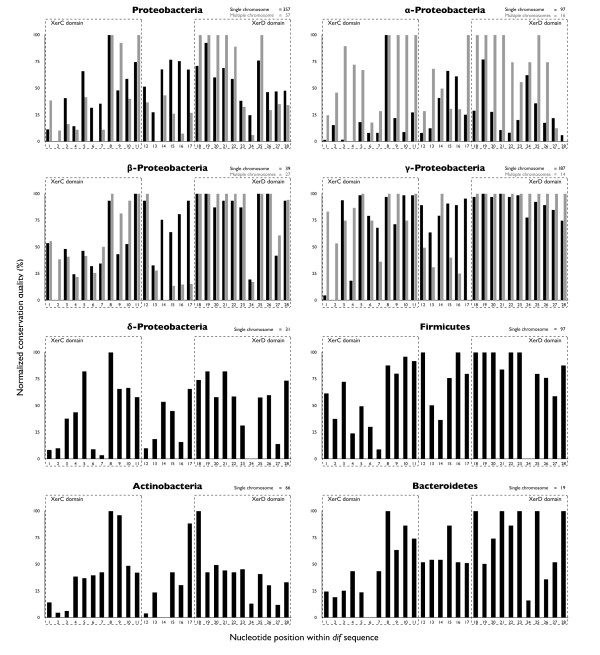
**The conservation of *dif *sequences**. This figure shows the conservation quantities at each position of *dif *sequence in each phylum or class (Proteobacteria, Firmicutes, Actinobacteria, Bacteroidetes, α-Proteobacteria, β-Proteobacteria, γ-Proteobacteria, and δ-Proteobacteria). The black bars represent the degree of conservation in single-chromosome genomes, and the gray bars represent that of organisms harboring multiple chromosomes. The labels "XerC domain" and "XerD domain" in these graphs represent the binding sites of these proteins. The X-axis represents the nucleotide positions in the *dif *sequence, and the Y-axis represents the nucleotide conservation quantity. Y-axis values were normalized to percentages.

Predicted *dif *sequences largely existed in non-coding regions (93.92%). More than half of these coding regions that contained *dif *sequences were hypothetical, with no functional annotation. Furthermore, we found two *dif *sequences included in phage ORF in *Vibrio *and *Xanthomonas*. While these sequences may be integrated with the phages by their hijacking of the host recombination machinery, these sequences are speculated to be the functional *dif *sites, due to 1. their unique occurrence within the genome opposite of the replication origin, and 2. their similarity as identified by our phylogenetic modeling approach. As previously shown in Proteobacteria [46], the XerC binding site is more variable and the XerD binding site is more conserved in all phyla (Figure 5), both for genomes with single chromosomes and for those with multiple chromosomes, presumably due to the interaction between XerD and FtsK for the initiation of first strand exchange [14]. The *dif *sequences in α-Proteobacteria with single chromosomes showed higher variation compared to these of other classes and phyla, but this variation was correlated with variations in genomic GC content (Additional file 1, Figure S4). These differences between variations are partly explains the failure of our prediction in extremely AT-rich genomes, such as those found in *C. perfringens *and *C. beijerinckii*.

Although *dif *sequences are expected to be located near the shift-point of the GC skew, we did not use this feature to predict and validate *dif *sequences with iterated HMM; therefore, using the comprehensively predicted *dif *sequences across numerous phyla, we were able to directly compare the positions of predicted *dif *sequences with those of the GC skew shift-points to analyze their relationships. As expected, these two positions are highly correlated in terms of genomic loci, confirming a previous work [38]. In this respect, because GC skew is the cumulative result of replicational selection/mutations, the degree of conservation of the CDR machinery is presumably in concordance with the degree of replication selection/mutation pressures (i.e. GC skew), which is partly characterized by the difference in the replication machinery and partly characterized by the growth rate [54]. On the other hand, as shown in Figure 2B, the differences in the positions of the GC skew shift-point and the strength of the GC skew, as quantified by GCSI, were not correlated. If replication termination occurs at the *dif *site, as proposed by Hendrickson and Lawrence [38], a stronger GC skew that is generated by a larger number of replication events and/or a higher mutation rate should statistically bring the GC skew shift-point closer to the *dif *site by the central limit theorem and law of large numbers. In fact, the overall correlation of these loci leads to the proposal that the *dif *site is the replication termination point. However, because a stronger degree of replication mutation/selection pressures does not bring these two loci closer to each other, they are not in a causal relationship. Therefore, although the *dif *sequence is located near the replication termination site for efficient CDR, the replication termination site is suggested to be at a site other than the *dif *site, as was recently shown *in vivo *[42]. On the other hand, the *dif *sequences in Firmicutes are more conserved in various phyla because the profile of Firmicutes was the best suited as the initial profile of iterated HMM in Chlorobi, Acidobacteria, Gemmatimonadetes, Nitrospirae, Elusimicrobia, Tenericutes, and Spirochaetes, where initial seed sequences were not available, and those in Proteobacteria were more variable, as shown by the requirement to predict by iterated HMM in classes instead of phyla. Tus proteins, which are shown to terminate replication *in vivo*, are more conserved in Proteobacteria and are not widely conserved in other, partly supporting the possible change in replication termination mechanism by a relatively recent takeover by the Tus-Ter system [43]. On the other hand, to the best of our knowledge, Tus analogues have not been comprehensively searched in other phyla, and therefore further analysis is required in order to fully support this hypothesis.

## Conclusions

By taking the phylogenetic iterated HMM approach and validating predicted candidates through a combination of HMMER score thresholds, conservation of palindromic structure, and cross-validation, we achieved a comprehensive identification of unique *dif *candidates in hundreds of genomes. As the result, we obtained unique candidate *dif *sequences in 715 chromosomes in 641 strains that were validated through multiple means, resulting in the largest collection of predicted *dif *sequences assembled to date. All of the predicted *dif *sequences described in this study, as well as visualizations of *dif *locations on circular genome maps, are freely available in an online database at http://www.g-language.org/data/repter/. The locations of *dif *sequences can be useful for studies of the regions surrounding the replication terminus, for phylogenetic studies of the replication termination and chromosome dimer resolution mechanisms, and can serve as supporting evidence for GC skew analyses.

Furthermore, we compared the positions of predicted *dif *sequences with those of the GC skew shift-points to understand the relationship between *dif *sequence and replication terminus using GCSI. As the result, although these two positions were highly correlated in terms of genomic loci, the differences in the positions of the GC skew shift-point and the GCSI were not correlated. Therefore, despite the *dif *sequence is located near the replication termination site for efficient CDR, the replication termination site is suggested to be at a site other than the *dif *site.

## Methods

### Software and sequences

All analyses in this study were conducted using programs written in Perl with the G-language Genome Analysis Environment, version 1.8.10 [55-57]. Hidden Markov Modeling and searching was conducted with HMMER, version 2.3.2 [45]. The *dif *sequence is the binding site of the XerCD recombinase; therefore, we first selected 734 circular bacterial chromosomes among 658 species/strains according to their conservation of XerCD using the KEGG (Kyoto Encyclopedia of Genes and Genomes) Orthology database (KO; [58]). We obtained these sequences from the NCBI FTP Repository [59]. The following experimentally confirmed (*E. coli *and *B. subtilis*) or computationally predicted (*F. alni*) *dif *sequences were used as seed sequences for subsequent searches and machine learning:

*E. coli *5'-GGTGCGCATAATGTATATTATGTTAAAT-3' [60]

*B. subtilis *5'-ACTTCCTAGAATATATATTATGTAAACT-3' [23]

*F. alni *5'-CACGCCGATAATGCACATTATGTCAAGT-3' [38]

### Iterated Hidden Markov Modeling

XerCD conservation does not immediately imply *dif *sequence conservation [20]. Therefore, to determine the phylogenetic conservation patterns of XerCD, we first aligned all XerCD amino acid sequences in the 734 genomes analyzed in this work with those in organisms with the above-mentioned *dif *sequences using ClustalW [61]. The average of the distances of XerC and XerD sequences that were calculated from this alignment were used to infer phylogenetic conservation patterns among phyla.

Based on the phylogenetic conservation patterns of XerCD, we iteratively created the hidden Markov models (HMM) for the accurate prediction of *dif *sequences, seeded with the previously described *dif *sequences (Figure 4A). Iterated HMM is shown to be able to build a more diverse and potentially more sensitive models than regular HMM, by incorporating distant homologous sequences while avoiding the contamination of non-homologous sequences into the model [62], and thus iterative HMM has been frequently utilized in bioinformatics and computational biology [63-66]. In this work, the first profile hidden Markov model was created from the *dif *sequences identified in genomes belonging in the same genus as the genome harboring the seed sequence. For example, in Proteobacteria, the seed sequences came from *E. coli; *therefore, the *dif *sequences were searched in 28 genomes belonging to the genus *Escherichia *by means of fuzzy matching with the seed sequences of *E. coli *K12 using Perl module String::Approx 3.26 [67]. For fuzzy matching, the maximum numbers of insertions, deletions, and substitutions were previously determined to be 0-bp, 0-bp, and 8-bp, respectively [60]. Likewise, initial profiles were created for Firmicutes based on 24 genomes in the genus *Bacillus *and for Actinobacteria based on two genomes in the genus *Frankia*. Based on these initial profile hidden Markov models, *dif *sequences were predicted in the genomes of the closest genus to the seed genus according to the amino acid sequences of XerCD proteins.

In the case of Proteobacteria, an initial profile was created using genomes belonging to the genus *Escherichia*, and this profile was used to predict *dif *sequences in the genus *Shigella*. Subsequently, a new profile was created using the previous profile and the newly predicted *dif *sequences, and this new profile was used to predict the second nearest genus (in the case of Proteobacteria, *Salmonella*). In this way, profile creation and *dif *sequence prediction were iterated in decreasing order of similarity of XerCD from the seed sequences; thus, iterated HMM was conducted for each phylum. Because no *dif *seed sequences were available for phyla other than the three described above, the three profile hidden Markov models obtained by iterated HMM in Proteobacteria, Firmicutes, and Actinobacteria were used as the initial profiles. At each iterated HMM, predicted candidates were validated according to the following criteria: 1) HMMER score ≥10 and E-value < 1.0e-04, 2) leave-one-out cross-validation using the new profiles, and 3) conservation of the palindromic structure. For cross-validation, each time a new profile was created in the iterated HMM, we tested the validity of the training set by leaving out one of the *dif *sequences from the accumulated set of *dif *sequences and checking that the prediction of the left-out sequence by training with all of the other *dif *sequences is always above the threshold for all *dif *sequences collected up to that iteration. For the palindromic structure, positions 7-12-bp and 17-22-bp of *dif *sequences, corresponding to the binding sites of XerC and XerD, were checked for complementarities. For example, the palindromic structure of *E. coli dif *sequences in bracket notation is "--(--- ((((((-()-)))))) ---)--", and the conservation threshold is set to more than four pairs of complementarities within the 7-12-bp and 17-22-bp positions of the predicted *dif *sequences.

Although iterated HMM is based on phyla, this taxonomic unit is sometimes too diverse to accurately follow phylogeny with recursive means. Therefore, prediction was separately conducted in classes instead of phyla for 60 strains, harboring 130 chromosomes for classes α-, β- and γ-Proteobacteria. Similarly, sometimes, a species is highly phylogenetically distant from the seed organism, making it the case that utilization of profile hidden Markov models from other phyla is more suitable than own phyla's profile. When iterated HMM fails in such cases, an alternative seed profile is created using the *dif *sequences from the top three genomes with the closest XerCD sequences, as determined by alignment using ClustalW (Figure 4B).

GC skew's shift-point, calculated as (C - G)/(C + G), was computed using the find_ori_ter function of the G-language GAE, based on the cumulative GC skew [68] at 1-bp resolution. Although GC skew is widely observed in bacterial species, a number of genomes do not exhibit notable compositional bias [48,69]. To determine the presence of genomic nucleotide compositional bias, the GC skew Index (GCSI) was calculated for all genomes, and GCSI ≥ 0.05 was used as the threshold [48,70]. GCSI quantifies the degree of GC skew using the compositional distance between the leading and lagging strands and the spectral amplitude of 1 Hz signal of GC skew graph using Fast Fourier Transform. In this study, the replication origin is defined based on the cumulative GC skew at 1-bp resolution using the G-language GAE [55].

### Calculation of the conservation quantity of *dif *sequences

Conservation quantity was calculated based on the nucleotide variance in each position of *dif *sequences in Figure 5. Firstly, we calculated the position-specific base composition of all *dif *sequences in a group (phylum or class). Subsequently, variance of the most frequent base in that position is calculated from the base composition. For example, when a group with 100 *dif *sequences has nth base composition of (A, T, G, C = 100, 0, 0, 0) or (A, T, G, C = 25, 25, 25, 25), the variance is 2500 or 0, respectively. Hence, if the position-specific base composition is biased toward any one base, its high variance indicates high degree of conservation. These values are normalized to percentages for comparison with other groups in Figure 5. In the case of multiple chromosomes, since these conservation quantities were calculated in each strain, the average value was used for normalization.

## Authors' contributions

NK carried out the analysis, and NK and KA wrote the manuscript. MT supervised the work, and all authors read and approved the final manuscript.

## Supplementary Material

Additional file 1 **AdditionalFigures.pdf**. Click here for file

Additional file 2 **Complete list of predicted *dif *sequences**. Click here for file
