# Supplementary material for: Comprehensive prediction of chromosome dimer resolution sites in bacterial genomes
Source: BMC Genomics. 2011 Jan 11;12:19. doi: 10.1186/1471-2164-12-19 (PMC3025954; doi:10.1186/1471-2164-12-19)
Supplement: Additional file 1 — AdditionalFigures.pdf. [file 1471-2164-12-19-S1.PDF]

Supplemental Figures

Figure S1 - An example for the plot shown in Figure 2

Figure S2 - Distribution of the genomic distance of *xerC*, *xerD* and *ftsK* gene from predicted *dif* sites

Figure S3 - The difference between *dif* and GC skew shift-point positions

Figure S4 - Variance of GC content distribution

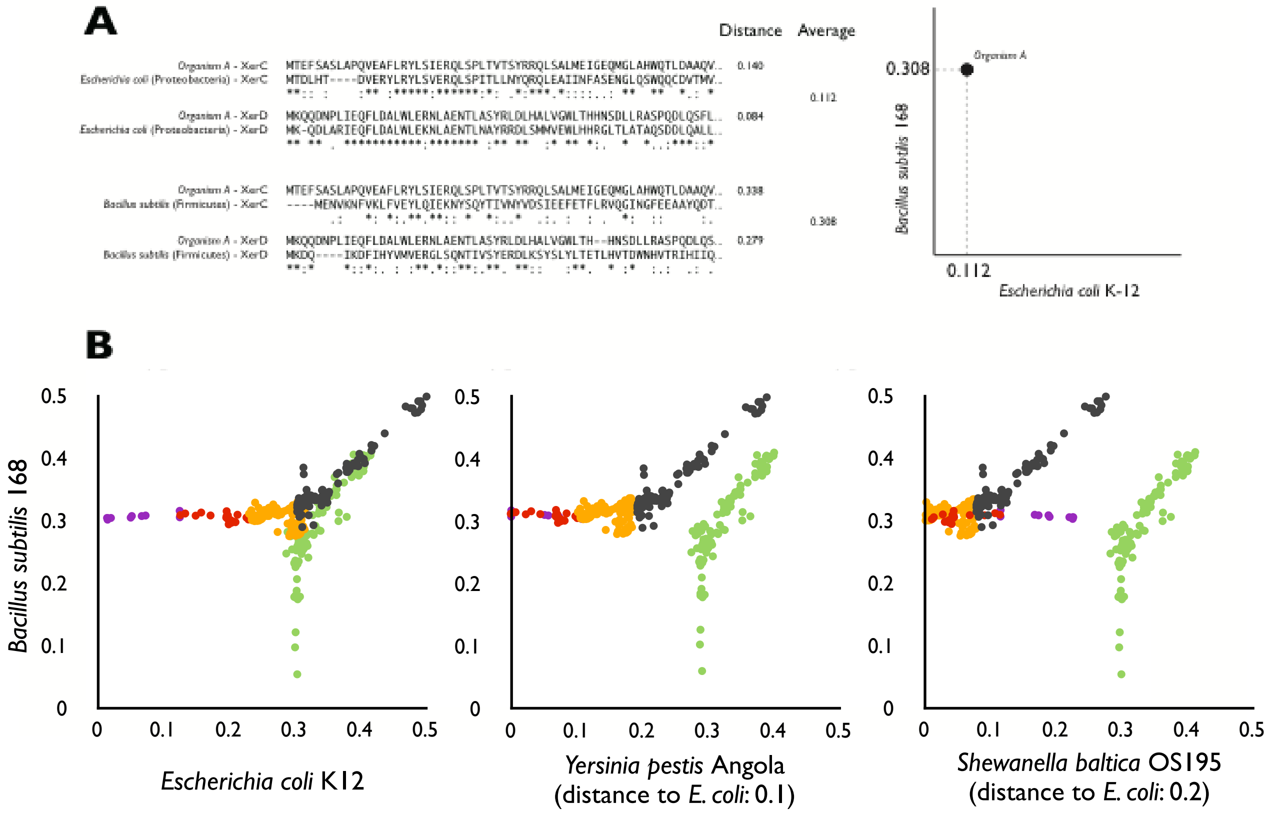

Figure S1 - An example for the plot shown in Figure 2

This phylogenetic distance was based on XerC and XerD amino acid sequence alignment in each other strain, and we calculated this distance using the average distance matrix that is generated from the pairwise scores. A: in this case of *organism A* in Proteobacteria, the average phylogenetic distances between *organism A* and *Escherichia coli* is 0.112, and that between *organism A* and *Bacillus subtilis* is 0.308. According to this scores, the *organism A* was plotted as the diagram. Since *organism A* belongs in Proteobacteria (represented as blue in Figure 2), it has shorter distance to *E.coli* than to *B. subtilis*. B: these graphs are shown by other spectrums. The left figure is Proteobacteria (blue) plot in Figure 2A, and colored in each distance (distance 0-0.1, 0.1-0.2, 0.2-0.3 and other are represented by purple, red, orange and gray respectively). The middle and right figures are used *Yersinia pestis* Angola (distance = 0.1) and *Shewanella baltica* OS195 (distance = 0.2) as X-axis respectively. The observation by such spectrum changes shows that the XerCD amino acid sequences are distinguished in each strain.

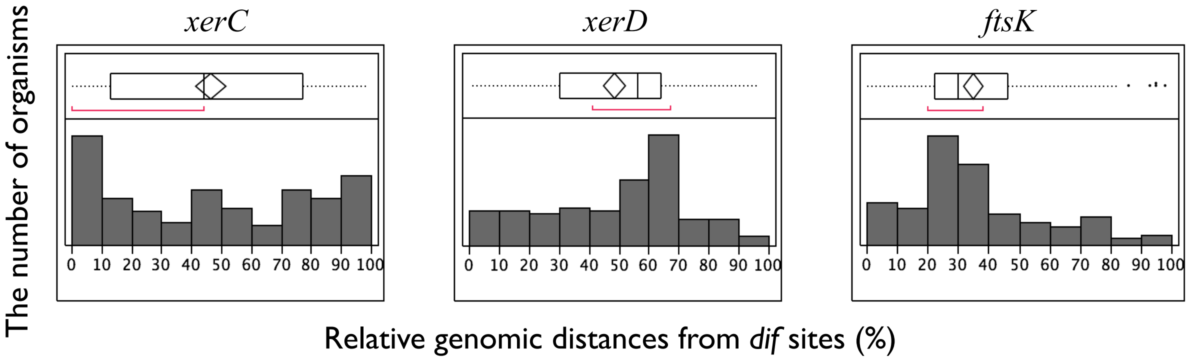

Figure S2 - Distribution of the genomic distances of *xerC*, *xerD* and *ftsK* gene from predicted *dif* sites

These histograms represent the distributions of relative genomic distances of *xerC*, *xerD* and *ftsK* genes from *dif* sites. X-axis represents the normalized genomic distance (%), and Y-axis represents the number of organisms in each group. The box plots represent the variance.

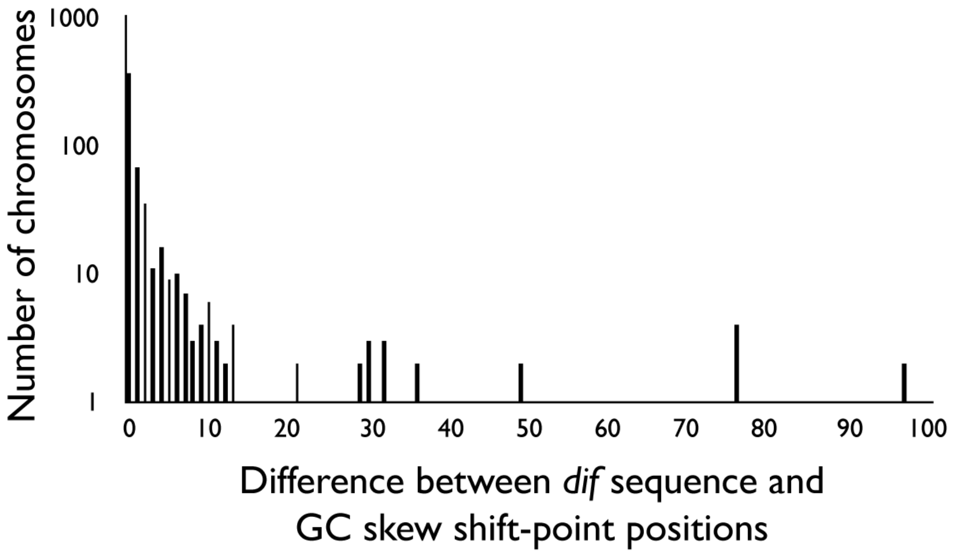

Figure S3 - The difference between *dif* and GC skew shift-point positions
The frequency distribution is in a logarithmic scale of the number of chromosomes grouped by the difference between the positions of the *dif* sequence and the GC skew shift-point.

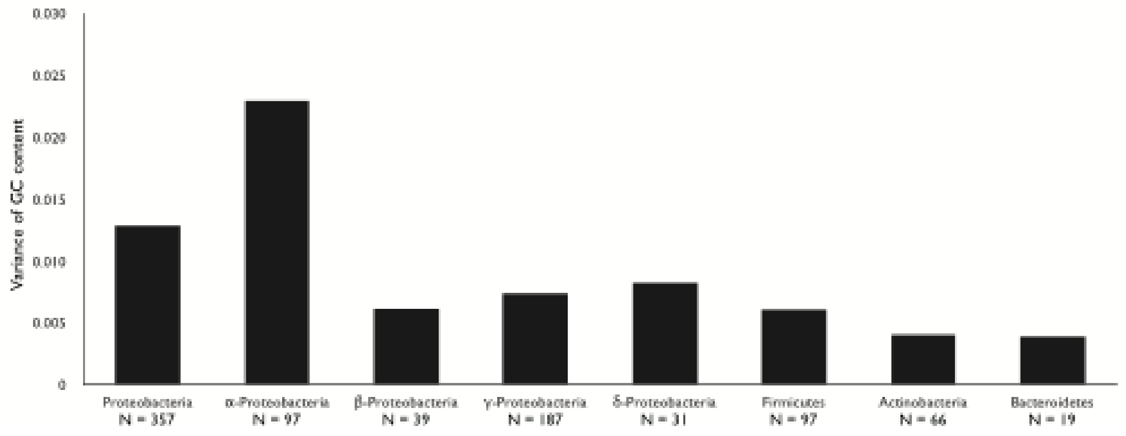

Figure S4 - Variance of GC content distribution

This graph shows the GC content variance of organisms in each phylum or class (Proteobacteria, Firmicutes, Actinobacteria, Bacteroidetes, α-Proteobacteria, β-Proteobacteria, γ-Proteobacteria, and δ-Proteobacteria). The X-axis represents the names of the phyla or classes and the number of included organisms. The Y-axis represents the variance of GC content.
